# Evidence for peripheral and central actions of codeine to dysregulate swallowing in the anesthetized cat

**DOI:** 10.3389/fneur.2024.1356603

**Published:** 2024-06-13

**Authors:** Donald C. Bolser, Tabitha Y. Shen, M. Nicholas Musselwhite, Melanie J. Rose, John A. Hayes, Teresa Pitts

**Affiliations:** ^1^Department of Physiological Sciences, University of Florida, Gainesville, FL, United States; ^2^Department of Speech, Language, and Hearing Sciences, Department of Biomedical Sciences, Dalton Cardiovascular Center, University of Missouri, Columbia, MO, United States

**Keywords:** swallow, codeine, breathing, airway, vagal reflexes

## Abstract

Systemic administration of opioids has been associated with aspiration and swallow dysfunction in humans. We speculated that systemic administration of codeine would induce dysfunctional swallowing and that this effect would have a peripheral component. Experiments were conducted in spontaneously breathing, anesthetized cats. The animals were tracheotomized and electromyogram (EMG) electrodes were placed in upper airway and chest wall respiratory muscles for recording swallow related motor activity. The animals were allocated into three groups: vagal intact (VI), cervical vagotomy (CVx), and supra-nodose ganglion vagotomy (SNGx). A dose response to intravenous codeine was performed in each animal. Swallowing was elicited by injection of 3 mL of water into the oropharynx. The number of swallows after vehicle was significantly higher in the VI group than in SNGx. Codeine had no significant effect on the number of swallows induced by water in any of the groups. However, the magnitudes of water swallow-related EMGs of the thyropharyngeus muscle were significantly increased in the VI and CVx groups by 2–4 fold in a dose-related manner. In the CVx group, the geniohyoid muscle EMG during water swallows was significantly increased. There was a significant dose-related increase in spontaneous swallowing in each group from codeine. The spontaneous swallow number at the 10 mg/kg dose of codeine was significantly larger in the CVx group than that in the SNGx group. During water-evoked swallows, intravenous codeine increased upper airway motor drive in a dose-related manner, consistent with dysregulation. The data support the existence of both central and peripheral actions of codeine on spontaneous swallowing. At the highest dose of codeine, the reduced spontaneous swallow number in the SNGx group relative to CVx is consistent with a peripheral excitatory action of codeine either on pharyngeal/laryngeal receptors or in the nodose ganglion itself. The higher number of swallows in the CVx group than the VI group supports disinhibition of this behavior by elimination of inhibitory vagal sensory afferents.

## 1 Introduction

Swallow is a critically important behavior for protecting the airways from aspiration in addition to its role in feeding. Swallowing is produced by a complex neural circuit that is functionally identified as the swallow pattern generator. This circuit is located in the brainstem, but is subject to significant modulation by suprapontine pathways (1). Further, swallowing also is modulated by peripheral sensory afferents from the lungs and airway (2, 3), chest wall (3), mouth, pharynx, and esophagus (1, 4–6) and these afferents travel through the vagus, trigeminal, or hypoglossal nerves.

Administration of opioids, including morphine and remifentanyl is associated with significant dysphagia and aspiration syndromes (7–11). The site(s) of opioid actions on swallowing are not fully understood. It is well known that systemic administration of opioids can activate pulmonary vagal afferents resulting in perturbation of laryngeal function (12, 13). Further, microinjection of opioids into the nodose ganglion can induce effects that are similar to activation of peripheral vagal afferents (14). Savilampi et al. (10) found that subjective and objective metrics of swallow were not affected by pretreatment with a peripherally-restricted opioid antagonist. The results of this study were consistent with a peripheral action of opioids to influence swallowing.

Codeine is one of the most commonly abused opioid drugs worldwide and is likewise among the most common pharmaceutical opioids found in fatal poisonings (15, 16). Systemic administration of this drug is known to stimulate vagal C-fibers to influence breathing (17). Further, codeine is well known to alter breathing and inhibit cough via central action (18–24). However, the influence of this opioid on swallowing is unknown. We speculated that intravenous codeine would have suppressive effects on swallow frequency and upper airway muscle EMG magnitudes and that some of these effects would be mediated by vagal afferents.

## 2 Methods

Experiments were performed on 19 spontaneously breathing adult male cats with an average age of 1.05 ± 0.03 years and weight of 4.95 ± 0.12 kg. These cats were purchased from Marshall BioResources (North Rose, NY) and pair-housed in the University of Florida Animal Care Services on a 12-h light/12-h dark cycle with food and water *ad libitum*. The protocol was approved by the University of Florida Institutional Animal Care and Use Committee (IACUC) and all procedures were compliant with the Guide for the Care and Use of Laboratory Animals. Anesthesia was initially induced with sevoflurane (4.5%), then the animals were weaned onto sodium pentobarbital (Lundbeck, Inc., Deerfield, IL, 25 mg/kg IV). Based on forelimb withdrawal reflex and jaw tone, supplemental doses were given as needed in 0.1–0.3 mL IV boluses. A single dose of atropine sulfate (0.054 mg/kg IV, Patterson Veterinary Supply, Inc., Devens, MA) was given at the beginning of surgery to minimize airway secretions. The femoral vein and artery were cannulated to deliver drugs and record blood pressure, respectively. Arterial blood pressure and end-tidal CO_2_ (30–38 mm Hg ETCO_2_; Datax Engstrom; Datax Ohmeda, Inc.; Madison, WI) were continuously recorded, and arterial blood gas samples (epoc Blood Analysis System; Siemens Healthineers USA) were obtained every hour. Body temperature was maintained at 37.5°C with a TC-1000 homeothermic pad and rectal temperature probe (CWE Inc.; United States). Esophageal pressure was measured via a small balloon inserted through the mouth into the midthoracic esophagus. The cats were euthanized by an overdose of pentobarbital (IV) followed by 3 mL of a saturated potassium chloride solution (IV) (Thermo Fisher Scientific, Waltham, MA). Animals were tracheostomized and allowed to breathe spontaneously.

Electromyograms (EMGs) were recorded using bipolar insulated fine wire electrodes (A-M Systems stainless steel #791050) according to the technique of Basmajian and Stecko (25). Eight muscles were used to evaluate swallow: mylohyoid, geniohyoid, thyrohyoid, thyropharyngeus, thyroarytenoid, upper esophageal sphincter (UES), parasternal, and costal diaphragm. The digastric muscles were dissected away from the surface of the mylohyoid and electrodes were placed on the left mylohyoid. A small horizontal incision was made at the rostral end of the right mylohyoid followed by an incision following the midline for approximately 1 cm to reveal the geniohyoid underneath. Electrodes were placed 1 cm from the caudal insertion of the right geniohyoid muscle. The thyroarytenoid electrodes were inserted through the cricothyroid window into the anterior portion of the left vocal fold, which were visually inspected post-mortem. Rotation of the larynx and pharynx counterclockwise revealed the superior laryngeal nerve, which facilitated placement of the left thyropharyngeus muscle electrodes. The thyropharyngeus is a fan shaped muscle with the smallest portion attached to the thyroid cartilage; electrodes were placed in the ventral, caudal portion of the muscle overlaying thyroid cartilage within 5 mm of the rostral insertion of the muscle. To place the electrodes within the cricopharyngeus muscle, the larynx and pharynx were rotated counterclockwise to reveal the posterior aspect of the larynx. The tissue was palpated for the edge of the cricoid cartilage and electrodes were placed just cranial to the edge of this structure (for a bilateral recording). The left thyrohyoid electrodes were inserted approximately 1 cm rostral to the attachment to the thyroid cartilage. The sternal diaphragm was placed by elevation of the sternum and the electrodes placed along the dorsal surface. Swallow was operationally defined as a large ballistic-like increase in the mylohyoid, geniohyoid, thyrohyoid, thyropharyngeus, and thyroartytenoid muscle EMGs in response to water swallow or codeine administration in conjunction with no increase in esophageal pressure. This definition separated swallow from other airway protective behaviors such as cough, sneeze, or expiration reflex which require increased intrathoracic pressure to be executed (20).

The positions of all electrodes were confirmed by visual inspection (following electrode placement and post-mortem) and by EMG activity patterns during breathing and swallow (6, 26–28).

### 2.1 Stimulation of swallow

Swallow was induced by infusing 3 ccs of water into the oropharynx via a 1-inch long, thin polyethylene catheter (outer diameter 0.5–1.0 mm). For all time points at least three trials were completed, separated by a minimum inter-stimulus interval of one minute. All water trials for each animal were performed by the same researcher to maintain stimulus consistency. Swallow number is the swallow count within 1 min after the water swallow stimulus. For codeine induced swallowing, swallow number was the total swallows that occurred within 1 min after the completion of the administration of codeine.

### 2.2 Animal groups

Animals were randomly assigned to one of three groups: (A) vagus intact (*n* = 5), (B) mid-cervical vagotomy (*n* = 7), and (C) supra-nodose vagotomy (*n* = 5). For cervical vagotomy, the vagosympathetic trunk was isolated from the surrounding tissue and sectioned bilaterally with small scissors at the level of the larynx. This intervention was intended to eliminate vagal afferent feedback from thoracic and abdominal organs. In supra-nodose vagotomy animals, 3–4 mm of the vagal trunk was isolated rostral to the jugular/nodose ganglionic complex by blunt dissection of the surrounding tissue. The supra-nodose vagal trunk was cut bilaterally with small scissors. This intervention was intended to eliminate sensory feedback from the larynx in addition to that from thoracic and abdominal organs. Laryngeal nerves directly enter the jugular/nodose ganglion complex, and this level is rostral to the site of section of the cervical vagus in the cervical vagotomy group.

### 2.3 Protocols

*Vagotomy.* To understand the impact of bilateral vagotomy swallow trials were completed just prior to and within 15 min post-vagotomy (mid-cervical or supra-nodose). There was at least a 30-min interval between the vagotomy and the start of the pharmacological trials.

*Codeine and vagotomy.* To additionally understand the impact of codeine, swallow trials were performed with a cumulative dose response to codeine monohydrate (C_18_H_21_NO_3_ · H_2_O; Sigma Aldrich Chemical Co. St. Louis, MO) infused intravenously. The protocol began a vehicle (0.9% saline) followed by a cumulative dose–response of 0.1, 0.3, 1, 3, 10 mg/kg controlling to total volume. All doses calculated as their free base and 10 min between each dose.

### 2.4 Analysis

EMG channels were rectified and smoothed with a 50 ms time constant prior to analysis.

The data was then normalized to the median amplitude following vehicle. All data are reported as a mean ± standard error (SEM) unless otherwise stated. Normality of the data was tested using the Kolmogorov–Smirnov normality test. For statistical analysis, two-way repeated-measures ANOVA (RM-ANOVA) was used to determine the effect of condition (vagotomy) and dose of codeine. Differences between swallow number pre- and post- cervical vagotomy and supra-nodose section were assessed with a paired *t*-test and a Wilcoxon matched-pairs signed-ranks test, respectively, using SigmaPlot (Grafiti LLC, Palo Alto, CA). Alpha was set with a *p*-value = 0.05.

## 3 Results

### 3.1 Swallow count

**Figure 1:**
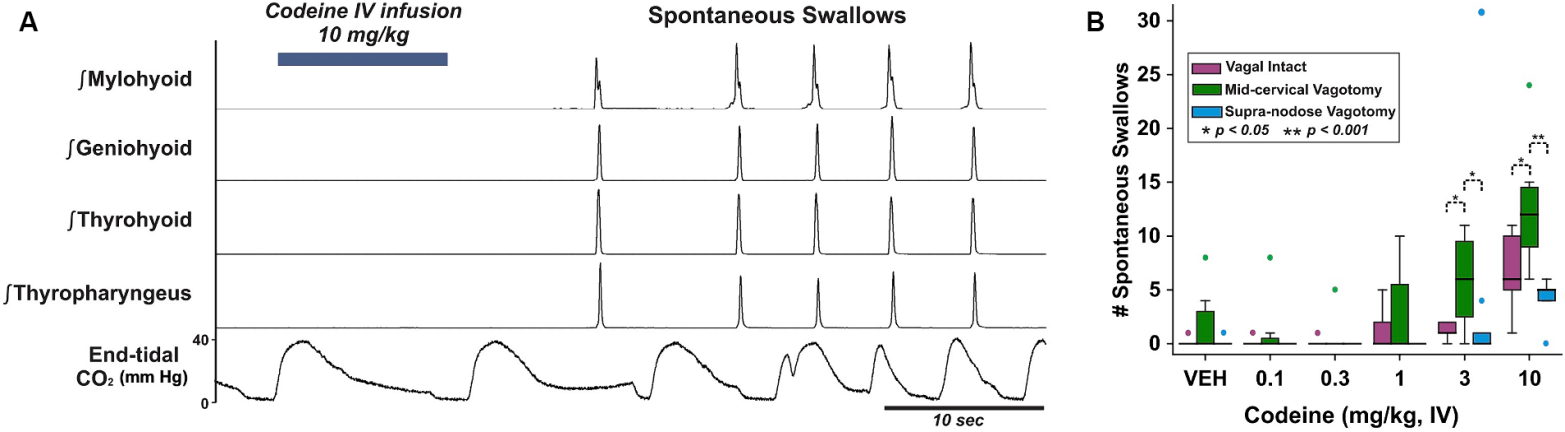
**(A)** Integrated upper airway muscle EMGs in a mid-cervical vagotomized cat after intravenous application of codeine and the resulting spontaneously produced swallows. Bottom, end-tidal CO_2_ oscillates with the breathing rhythm and is disrupted by swallows. **(B)** Box-and-whisker plots show that spontaneously produced swallows increased with mid-cervical vagotomy but not supra-nodose vagotomy. VEH, vehicle; vagus intact (*n* = 5), mid-cervical vagotomy (*n* = 7), and supra-nodose vagotomy (*n* = 5). Colored markers are outliers for each condition.

Swallows occurring without an oropharyngeal stimulus (i.e., spontaneous) are very rare in this preparation, however they were observed consistently in response to codeine (Figure 1A). A two-way repeated measures ANOVA was performed to evaluate the effects of vagotomy and codeine administration on number of spontaneous swallows (Figure 1B). The results indicated a significant main effect for vagal condition [RM-ANOVA: *F*(2, 70) = 3.99, *p* = 0.04] and dose of codeine [*F*(5, 70) = 14.58, *p* < 0.001]. Student–Newman–Keuls post-hoc testing revealed an effect condition at 3 and 10 mg/kg with significantly more spontaneous swallows in the mid-cervical group compared to vagal intact animals [3 mg/kg, *q* = 3.33, *p* = 0.003; 10 mg/kg, *q* = 2.76, *p* = 0.02] and animals with a supra-nodose vagotomy [3 mg/kg, *q* = 3.42, *p* = 0.004; 10 mg/kg, *q* = 3.94, *p* < 0.001].

In response to the water stimuli there was a significant reduction in average number of swallows produced following mid-cervical (*t*-test: 2.1 ± 0.4 pre to 1.1 ± 0.3 post; *p* = 0.006) and supra-nodose (Wilcoxen test: 4.0 ± 1.0 pre to 0.8 ± 0.5 post; *p* = 0.03) vagotomy (Table 1). Supra-nodose vagotomy resulted in so few swallows that our analysis could not reliably determine how the swallow motor pattern was affected by the procedure.

**Table 1:** Swallow counts induced by water under vagus intact, cervical vagotomy, and supra-nodose vagotomy conditions.

	Codeine doses						
Vagus intact (*n* = 5)	Pre-cut	VEH	0.1 mg/kg	0.3 mg/kg	1.0 mg/kg	3.0 mg/kg	10.0 mg/kg
Water swallow count	n.a. ± n.a.	2.7 ± 0.7^*,†^	2.6 ± 0.7^**^	2.1 ± 0.5^*^	1.6 ± 0.3	1.4 ± 0.4	1.8 ± 0.3^*^
Cervical vagotomy (*n* = 7)	Pre-cut	VEH	0.1 mg/kg	0.3 mg/kg	1.0 mg/kg	3.0 mg/kg	10.0 mg/kg
Water swallow count	2.1 ± 0.4	1.1 ± 0.3	1.3 ± 0.3	1.7 ± 0.4^*^	1.5 ± 0.3	1.8 ± 0.3^*^	1.7 ± 0.4^*^
Supra-nodose Vagotomy (*n* = 5)	Pre-cut	VEH	0.1 mg/kg	0.3 mg/kg	1.0 mg/kg	3.0 mg/kg	10.0 mg/kg
Water swallow count	4.0 ± 1.0	0.8 ± 0.5	0.4 ± 0.3	0.2 ± 0.2	0.6 ± 0.5	0.3 ± 0.2	0.3 ± 0.1

Values reported are mean ± SEM. Statistical significance relative to SNVx at a given dose is indicated by ^*^p < 0.05 or ^**^p < 0.01. Statistical significance relative to CVx at a given dose is indicated by ^†^*p* < 0.05. VEH, vehicle. SNVx, supra-nodose vagotomy. CVx, mid-cervical vagotomy. n.a., not applicable.

### 3.2 Swallow motor pattern

**Figure 2:**
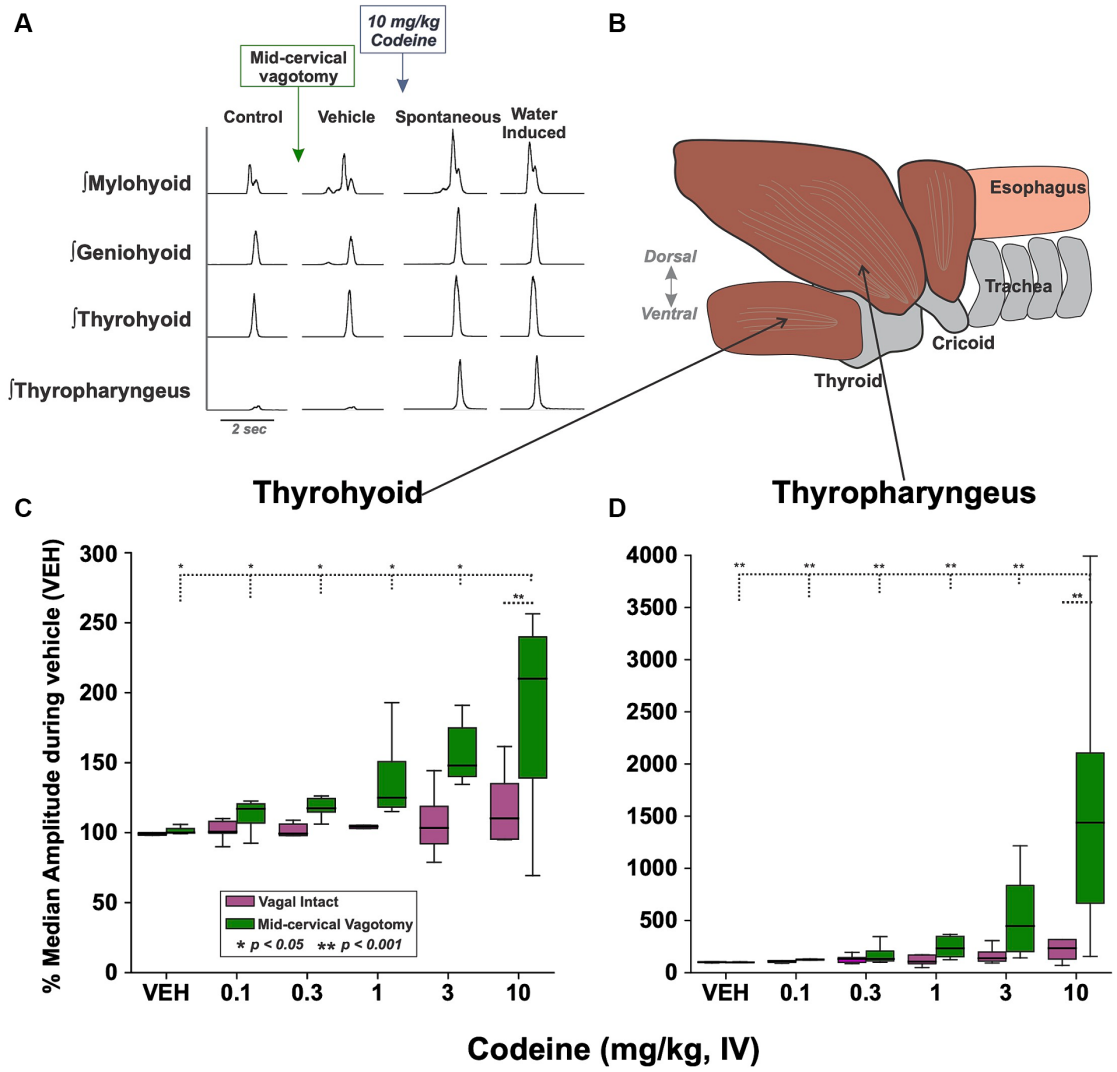
**(A)** Examples of upper airway muscle EMGs showing swallowing behavior before/after mid-cervical vagotomy (left) and before/after intravenous codeine application (middle). Approximately 1 min after the presentation of each codeine dose water trials were begun (right). **(B)** A sagittal view showing the muscle arrangement of the thyrohyoid **(C)** and thyropharyngeus **(D)** muscles. Box plots show that EMG amplitude increased with codeine doses under the mid-cervical vagotomy condition for both muscles. VEH, vehicle.

EMG amplitude from submental muscles (mylohyoid and geniohyoid), an extrinsic laryngeal muscle (thyrohyoid) and the inferior pharyngeal constrictor (thyropharyngeus) were compared (Figures 2A,B) to determine the effect of mid-cervical vagotomy and codeine using 2-way ANOVA. There was no significant change to the maximum EMG amplitude during water swallow of the *mylohyoid* [condition (*p* = 0.2) or dose (*p* = 0.5)] or *geniohyoid* [condition (*p* = 0.1) or dose (*p* = 0.3)] (Table 2).

**Table 2:** Changes in EMG amplitudes for water-induced swallows relative to vehicle for upper airway muscles under vagus intact and cervical vagotomy conditions.

Vagus intact (*n* = 5)	Codeine doses						
Water swallow amps. (% relative to VEH)	Pre-cut	VEH	0.1 mg/kg	0.3 mg/kg	1.0 mg/kg	3.0 mg/kg	10.0 mg/kg
**Hyoid/Laryngeal elevators**							
Mylohyoid	n.a. ± n.a.	101 ± 4	100 ± 9	106 ± 10	98 ± 13	83 ± 11	108 ± 45†
Geniohyoid	n.a. ± n.a.	96 ± 2	93 ± 16	100 ± 19	95 ± 30	91 ± 41	121 ± 49††
Thyrohyoid	n.a. ± n.a.	106 ± 17	101 ± 8	98 ± 12	104 ± 15	107 ± 28	119 ± 29††
**Pharyngeal**							
Thyropharyngeus	n.a. ± n.a.	101 ± 5	118 ± 32	132 ± 43	117 ± 53	170 ± 96	294 ± 254†††
Cervical vagotomy (*n* = 7)	Codeine doses						
Water swallow amps. (% relative to VEH)	Pre-cut	VEH	0.1 mg/kg	0.3 mg/kg	1.0 mg/kg	3.0 mg/kg	10.0 mg/kg
**Hyoid/Laryngeal elevators**							
Mylohyoid	145 ± 148	106 ± 10	146 ± 83	132 ± 71	151 ± 84	202 ± 153	934 ± 1878†
Geniohyoid	88 ± 31	106 ± 12^*^	120 ± 53^*^	126 ± 45^*^	143 ± 40^*^	189 ± 99^*^	547 ± 802††
Thyrohyoid	126 ± 58	110 ± 25^*^	124 ± 36^*^	120 ± 12^*^	139 ± 31^*^	156 ± 70^*^	262 ± 243††
**Pharyngeal**							
Thyropharyngeus	151 ± 87	103 ± 8^***^	122 ± 14^***^	175 ± 96^***^	303 ± 221^***^	555 ± 445^***^	1,590 ± 1,305†††

Values reported are mean ± SEM. VEH, vehicle. n.a., not applicable. Statistical significance relative to 10 mg/kg codeine of other doses, under the given vagal condition, is indicated by *p < 0.05 or ***p < 0.001. Statistical significance for a muscle EMG between vagal conditions at a given dose is indicated by ^†^*p* < 0.05, ^††^*p* < 0.01 or ^†††^*p* < 0.001.

For the *thyrohyoid* (Figure 2C) there was a significant effect of vagal condition [RM-ANOVA: *F*(1, 57) = 4.87, *p* = 0.03]. At 10 mg/kg thyrohyoid EMG amplitude was significantly greater following mid-cervical vagotomy (*q* = 4.05, *p* = 0.006) compared to vagus intact animals. For the *thyropharyngeus* (Figure 2D) there was a significant effect of vagal condition [*F*(1, 57) = 8.09, *p* = 0.006] and codeine dose [*F*(5, 57) = 5.69, *p* < 0.001]. For condition, at 10 mg/kg thyropharyngeus EMG amplitude was significantly greater following mid-cervical vagotomy (541%, *q* = 6.82, *p* < 0.001) compared to vagus intact animals. For dose, post-hoc testing revealed no change in EMG magnitude with codeine in vagally intact animals, however post mid-cervical vagotomy the EMG magnitude at 10 mg/kg was significantly greater than at 0.1 (*q* = 8.47, *p* < 0.001), 0.3 (*q* = 7.83, *p* < 0.001), 1 (*q* = 7.13, *p* < 0.001), and 3 (*q* = 5.97, *p* < 0.001) mg/kg.

## 4 Discussion

The major findings of this study were that intravenous codeine induced spontaneous swallowing in a dose-dependent manner in vagal intact and vagally denervated cats. This drug did not increase the frequency of water-induced swallows in any group. During water swallows, codeine increased the EMG magnitudes of selected upper airway muscles (geniohyoid, mylohyoid, thyrohyoid and thyropharyngeus) only in animals with mid-cervical vagotomy.

To our knowledge, this is the first report that the mu-opioid receptor agonist, codeine, alters swallow function in an animal model. In humans, the mu-opioid receptor agonist, remifentanil, altered pharyngeal function and esophageal motility during swallowing (7–11, 29). These effects included obvious aspiration (9), reductions in pharyngeal bolus movement (11), altered upper esophageal sphincter function (7), and a reduction in the strength of pharyngeal contractions (11). Similar effects were observed with morphine (11, 30). The effects of remifentanil on pharyngeal swallowing were not attenuated following administration of a peripherally acting derivative of the opioid antagonist naltrexone (7), suggesting a central action of the opioid. We are not aware of any report in the literature that codeine alters swallow function on the human. We note that we observed enhancement of pharyngeal swallow at doses of this drug (3 and 10 mg/kg IV) that were frankly respiratory depressant in this model (data not shown). In the human, prescription oral doses of codeine typically do not result in respiratory depression. It is possible that codeine in the human would have significant effects on swallow function at higher doses or if administered by the intravenous route.

We cannot attribute these effects of codeine solely to its actions at mu-opioid receptors given that it is a nonspecific drug. Codeine binds to an allosteric site on the central nicotinic receptor and enhances cholinergic transmission by a mechanism that is not sensitive to the alpha-7 nicotinic receptor antagonist methyllycaconitine (31–33). Therefore, the excitatory effects on pharyngeal swallowing observed in this study may be specific to codeine and not extendable to mu-opioid agonists in general.

Codeine induced swallowing in the absence of a peripheral stimulus, and swallow number was higher in vagotomized relative to vagal intact or SNGx animals. These observations support several different conclusions. First, codeine can induce swallowing in animals that have vagal axons and/or vagal axons plus ganglia sectioned, indicating that the swallow-promoting actions of this drug do not require sensory feedback from vagal sources to occur. Second, under vagus intact conditions sensory feedback blunted the swallow-inducing effects of codeine. Third, the differences between cervical vagotomy and section of the vagal trunk rostral to the nodose ganglion support the concept that ganglion cells and/or superior laryngeal nerve afferent pathways had a role in blunting the actions of codeine. The superior and inferior laryngeal nerves enter the vagal ganglion complex and this anatomical site is rostral to the typical location of cervical vagotomy. This afferent pathway was intact in our CVx animals, but was eliminated in the SNGx group.

While codeine is well-known to have central actions (18, 34), it and other opioids also alter the excitability of thoracic vagal and perhaps laryngeal afferents (13, 17, 35). The extent to which the swallow-inducing actions of codeine were due to solely central effects of this drug that were modified by spontaneous discharge of vagal sensory pathways or were due to both central and peripheral actions is unknown.

Codeine had differential actions on swallowing when actuated by increasing doses of the drug itself or a water stimulus. Spontaneous swallow count increased in a dose-dependent manner in response to the drug (Figure 1B), especially in CVx animals, yet the swallow count did not increase when the behavior was elicited by water (Table 1). First, codeine may have acted to decrease the threshold for actuation of swallow, presumably at the central level. Second, codeine may have acted on the swallow central pattern generator for swallowing if it is subordinate to a distinct threshold mechanism. Third, this drug may have increased the excitability of other circuits that stimulate swallowing through disinhibition. For example, Pitts and coworkers have shown that spinal pathways can have significant excitatory effects on swallowing (36). Our approach cannot separate between these three hypotheses and they are not mutually exclusive.

These findings also support the concept that swallow frequency can be controlled differently than motor drive to upper airway muscles. In response to water stimuli, swallow number did not change but the magnitudes of upper airway muscle EMGs significantly increased, especially in CVx animals. As noted above, enhancement of motor drive to selected upper airway muscles during swallowing can be observed following cervical spinal injury (36). The extent to which this mechanism can be actuated by drugs in the absence of spinal injury is unknown.

We conducted these experiments solely in male animals. Recently, Huff et al. (3) showed in a mouse model that there were no differences in swallow number or upper airway muscle EMG magnitudes between sexes under control conditions in vagal intact animals. However, male mice had 30–40% higher motor drive to geniohyoid and mylohyoid muscles after vagotomy. In our study, cervical vagotomy did not significantly alter motor drive to these two muscle groups or the thyrohyoid or thyropharyngeus muscles during water swallow but post vagotomy magnitudes were highly variable. The extent to which vagotomy alters upper airway muscle motor drive in female cats is unknown.

Bautista and co-workers have shown that swallowing can occur during autoresuscitation (37) and this behavior has been proposed to be important in the autoresuscitation process (38). It is likely that the role of swallowing in autoresuscitation is one of oropharyngeal clearance. Motor activation of pharyngeal muscles to move material out of the upper airway is an essential component of this behavior. Given that codeine initiated spontaneous swallowing but did not increase swallow number after water swallows, the action of this drug may be on neural elements that participate in non-ingestive functions of swallow control, such as autoresuscitation.

There were very large magnitude water swallows in our study for some upper airway muscle EMGs. This effect was magnified somewhat by the fact that vagotomy reduced EMG magnitudes for the thyropharyngeus in four animals. Although this effect was not statistically significant it did alter normalization. However, the largest water swallows in these animals were still several hundreds of percent larger than pre-vagotomy levels. As such, we chose not to remove these swallows from the dataset as outliers. It is our view that others who may choose to repeat our work be informed regarding the large range of swallow EMG magnitudes that can be observed after administration of codeine.

In conclusion, intravenous codeine induced spontaneous swallowing in the anesthetized cat. Further, the magnitudes, but not the frequency of occurrence of water-induced swallows were enhanced by this drug. These effects were larger in vagotomized animals. The results support a central action of codeine to enhance swallowing in the anesthetized cat model. Mid-cervical vagotomy resulted in a greater stimulation of spontaneous swallowing than in vagal intact animals. We suggest that systemic administration of codeine also actuated vagal afferent reflexes that suppress the expression of centrally activated spontaneous swallowing. The receptor-based mechanism for this effect may be more related to binding of codeine to an allosteric site on nicotinic receptors than its binding affinity for mu-opioid receptors. The central neural elements responsible for these effects of codeine are unknown.

## Data availability statement

The raw data supporting the conclusions of this article will be made available by the authors, without undue reservation.

## Ethics statement

The animal study was approved by University of Florida Institutional Animal Care and Use Committee. The study was conducted in accordance with the local legislation and institutional requirements.

## Author contributions

DB: Conceptualization, Data curation, Formal analysis, Funding acquisition, Methodology, Project administration, Resources, Supervision, Validation, Writing – original draft, Writing – review & editing. TS: Data curation, Formal analysis, Investigation, Methodology, Writing – original draft. MM: Investigation, Methodology, Writing – review & editing. MR: Investigation, Methodology, Writing – review & editing. JH: Writing – review & editing. TP: Data curation, Formal analysis, Investigation, Writing – review & editing.
